# Early Insights from Metabolic Dysfunction-Associated Steatotic Liver Disease (MASLD) Patients: An Observational Study on Polygenic Risk and Liver Biomarkers

**DOI:** 10.3390/ijms26178426

**Published:** 2025-08-29

**Authors:** Pietro Torre, Benedetta Maria Motta, Tommaso Sarcina, Mariano Festa, Mario Masarone, Marcello Persico

**Affiliations:** Department of Medicine, Surgery and Dentistry, “Scuola Medica Salernitana”, University of Salerno, Via S. Allende, 84081 Baronissi, Italy; ptorre@unisa.it (P.T.); tsarcina@unisa.it (T.S.); mafesta@unisa.it (M.F.); mmasarone@unisa.it (M.M.); mpersico@unisa.it (M.P.)

**Keywords:** metabolic dysfunction-associated steatotic liver disease, polygenic risk score, liver fibrosis

## Abstract

Metabolic dysfunction-associated steatotic liver disease (MASLD) is a growing public health concern influenced by both genetic and metabolic factors. Polygenic risk scores (PRSs), which combine the effects of known single-nucleotide polymorphisms (SNPs), may improve early risk stratification. We conducted an observational study on 298 MASLD patients: 148 from a Hepatology Unit and 150 from a Bariatric Surgery Unit. Genotyping was performed for the *PNPLA3*, *TM6SF2*, *MBOAT7*, and *GCKR* variants. A PRS was calculated and used to stratify patients by genetic risk. Liver fibrosis was assessed using the FIB-4 index, and a subset also underwent transient elastography. Clinical, biochemical, and anthropometric data were analyzed across genetic strata. PRSs showed positive correlations with AST, ALT, and FIB-4, indicating increased liver injury and fibrosis risk with higher genetic burden. Transaminases increased significantly across PRS quartiles (*p* < 0.05), and individuals with PRS > 0.532 exhibited elevated AST, ALT, and borderline FIB-4. Variant-specific associations included *PNPLA3* with increased AST and *MBOAT7* with higher hepatic steatosis (CAP). Subgroup analyses revealed distinct genetic and phenotypic patterns between the two clinical cohorts. These findings support the additive role of genetic risk in MASLD progression and underscore the value of polygenic profiling for the early identification and personalized management of high-risk patients.

## 1. Introduction

Metabolic dysfunction-associated steatotic liver disease (MASLD), formerly non-alcoholic fatty liver disease (NAFLD), encompasses a wide clinical spectrum ranging from benign steatosis to cirrhosis and hepatocellular carcinoma [1,2]. Its increasing prevalence reflects the global rise in metabolic syndrome and obesity. Fibrosis remains the principal determinant of liver-related morbidity and mortality [3]. While non-invasive scores such as FIB-4 and liver stiffness (LS) measured by elastography have improved risk stratification, they lack sensitivity in the obese population and in early disease stages, showing a lower sensitivity for detecting mild fibrosis compared to more advanced stages [4].

Global MASLD prevalence is estimated to be from 25% to over 40% among adults worldwide, with increasing incidence due to the obesity and type 2 diabetes epidemics [5]. MASLD affects both genders, is slightly more common in males, and predominantly presents in middle-aged adults. Clinical manifestations often include discomfort in the right upper quadrant, fatigue, and decreased health-related quality of life, even if many patients remain asymptomatic until advanced fibrosis develops [6,7]. Risk factors include obesity, insulin resistance, dyslipidemia, and a sedentary lifestyle. Moreover, genetic predisposition significantly influences MASLD development and progression. Notable single nucleotide polymorphisms (SNPs), including PNPLA3 rs738409, TM6SF2 rs58542926, MBOAT7 rs641738, and GCKR rs1260326, are consistently linked to steatosis, inflammation, and fibrosis in MASLD [8,9,10,11,12,13,14]. Polygenic risk scores (PRS), which aggregate risk across multiple variants, have emerged as promising tools to stratify disease risk. Their clinical utility in predicting MASLD severity across patient populations remains to be fully explored [12,15,16].

While obesity is a well-established risk factor for MASLD, patients in internal medicine settings may present with metabolic comorbidities even in the absence of extreme obesity. Current interventions focus on lifestyle modification, metabolic optimization, and emerging pharmacotherapies, yet early identification of high-risk individuals remains challenging. To address this gap, our study aimed to explore and integrate genetic, clinical, and non-invasive markers to improve MASLD stratification and explore population-specific variations between hepatology-referred and bariatric surgery patients.

## 2. Results

### 2.1. Characteristics of the Study Population

A total of 298 individuals with MASLD were included in the study, comprising two distinct clinical cohorts: Cohort 1 (n = 148), representing patients from an internal medicine setting, and Cohort 2 (n = 150), consisting of patients undergoing bariatric surgery. Demographic, anthropometric, and biochemical parameters are summarized in Table 1.

Patients in Cohort 1 were significantly older than those in Cohort 2 (*p* = 2.7 × 10^−19^) and had a lower proportion of females. As expected, body mass index (BMI) was significantly higher in Cohort 2 (41.7 ± 4.6 kg/m^2^) compared to Cohort 1 (33.1 ± 5.3 kg/m^2^, *p* = 3.9 × 10^−24^), reflecting the obesity-oriented inclusion criteria for the bariatric subgroup.

### 2.2. Liver Enzymes and Fibrosis Index

**Figure 1:**
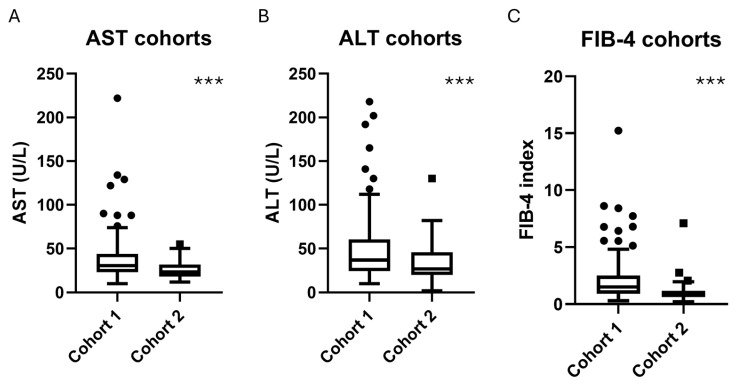
Distribution of liver function markers AST, ALT, and the fibrosis index FIB-4 across the patients’ cohorts. Boxplots represent the median, interquartile range (IQR), and range of (**A**) AST levels, (**B**) ALT levels, and (**C**) FIB-4 scores for the Internal Medicine cohort and the Obesity Surgery cohort. Differences between cohorts were assessed using Mann–Whitney U test (*** *p* values ≤ 0.005). N = 140 for Cohort 1, n = 68 for Cohort 2. Abbreviations: AST (Aspartate Aminotransferase), ALT (Alanine Aminotransferase), FIB-4 (Fibrosis-4 index). Outlier points represent extreme values outside interquartile ranges.

Liver enzymes differed significantly between groups. AST levels were higher in Cohort 1 (38.4 ± 26.6 U/L) than in Cohort 2 (25.8 ± 9.9 U/L, *p* = 1.3 × 10^−5^), as were ALT values (48.3 ± 36.9 vs. 34.7 ± 22.1 U/L, *p* = 0.005). Importantly, FIB-4 index, a validated non-invasive marker of hepatic fibrosis, was significantly higher in Cohort 1 compared to Cohort 2 (2.09 ± 1.98 vs. 1.02 ± 0.87, *p* = 4.0 × 10^−5^), suggesting more advanced liver damage in the metabolically compromised group (Figure 1).

Although liver stiffness, measured by transient elastography, was numerically greater in Cohort 1 (10.3 ± 11.2 kPa) than in Cohort 2 (8.3 ± 7.9 kPa), this difference did not reach statistical significance.

### 2.3. Hepatic Steatosis and Genetic Risk

Controlled attenuation parameter (CAP) values, indicative of hepatic steatosis, tended to be higher in Cohort 2 (312.5 ± 39.4 dB/m) compared to Cohort 1 (302.0 ± 43.7 dB/m, *p* = 0.084), in line with the greater degree of obesity observed.

**Figure 2:**
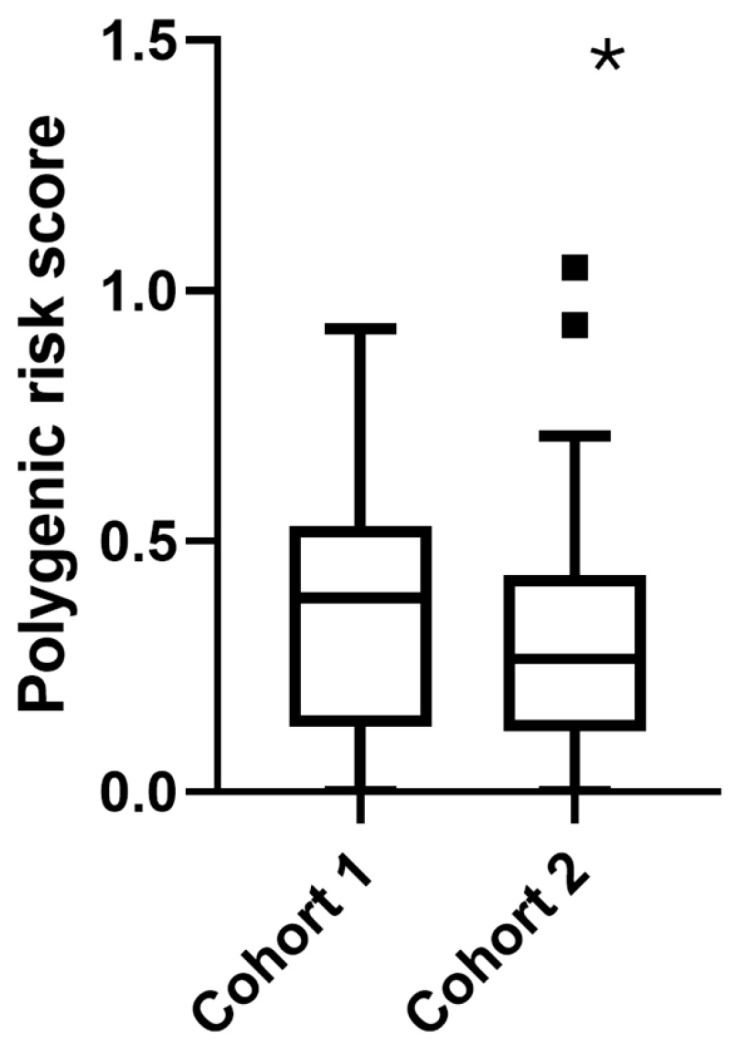
Comparison of polygenic risk scores (PRSs) between patient cohorts. Boxplot depicting the distribution of PRS in Cohort 1 (Internal Medicine) and Cohort 2 (Obesity Surgery). The PRS difference between cohorts was assessed using Mann–Whitney U test (* *p* values < 0.05). N = 142 for Cohort 1, n = 88 for Cohort 2. Outlier points represent extreme values outside interquartile ranges.

Additionally, polygenic risk score (PRS)—used as a marker of inherited predisposition to liver disease—was modestly higher in Cohort 1 than in Cohort 2 (0.368 ± 0.24 vs. 0.302 ± 0.21, *p* = 0.047; Figure 2, although nominal significance was lost after Bonferroni’s correction), suggesting a greater genetic contribution to liver damage in the internal medicine group.

These findings delineate two clinically and pathophysiologically distinct MASLD subgroups: a metabolically high-risk population (Cohort 1) with increased fibrosis burden and genetic susceptibility, and a predominantly obese cohort (Cohort 2) with more pronounced steatosis but lower fibrosis scores.

### 2.4. Total Cohort Analysis

Across the entire MASLD cohort, the polygenic risk score (PRS) correlated positively with AST, ALT, and the FIB-4 index (Table 2). Using the conservative Bonferroni method of adjustment for multiple comparisons, we obtain a *p*-value significance cut-off of 0.0083 instead of 0.05. A statistically significant correlation is still supported in the presence of the adjustment.

Liver stiffness (LS) showed significant associations with AST, CAP, and FIB-4 (all *p* < 0.005), and a trend toward significance for ALT (*p* = 0.081).

**Figure 3:**
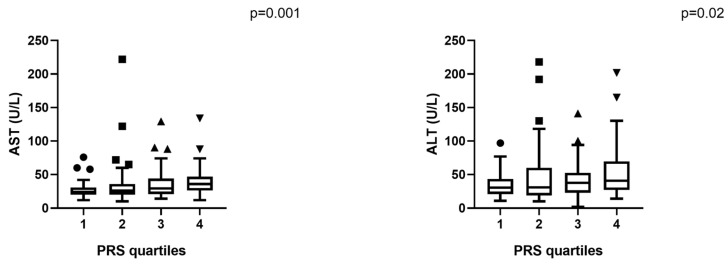
Stratification of liver biomarkers by PRS quartiles. Box plots comparing AST and ALT across PRS quartiles in the total population. Median values rise from Q1 to Q4 (Kruskal–Wallis, *p* < 0.05). Abbreviations: AST (Aspartate Aminotransferase), ALT (Alanine Aminotransferase). Outlier points (circles, squares, and triangles) represent extreme values outside interquartile ranges.

When stratified by PRS quartiles, higher genetic risk was associated with increased AST and ALT levels (*p* < 0.05; Figure 3). In the high-risk PRS group (PRS > 0.532), AST levels were significantly elevated (*p* < 0.005), as well as ALT levels (*p* = 0.025), with a borderline increase in FIB-4 (*p* = 0.055).

Fibrosis risk categories (as assessed by FIB-4 thresholds) increased progressively with higher PRS and LS values (*p* < 0.05).

### 2.5. Cohort-Specific Analysis

#### 2.5.1. Cohort 1: Internal Medicine

In the Hepatology cohort, PRS significantly correlated with AST, ALT (*p* < 0.005), and FIB-4 (*p* = 0.04). LS was strongly associated with AST, ALT, CAP, and FIB-4 (*p* < 0.05 for all).

AST (*p* = 0.006) and ALT (*p* = 0.019) levels increased across PRS quartiles, and AST was significantly elevated in individuals with PRS > 0.532 (*p* = 0.047).

Fibrosis risk increased with higher LS (*p* < 0.05).

#### 2.5.2. Cohort 2: Bariatric Surgery

In the Bariatric Surgery cohort, PRS was significantly associated with FIB-4 (*p* = 0.044). CAP correlated with LS and was significantly associated with both AST (*p* = 0.012) and ALT (*p* = 0.001).

Notably, triglyceride (TG) levels decreased progressively across PRS quartiles (*p* = 0.022), suggesting an inverse relationship between genetic liver risk and circulating lipids in this group.

### 2.6. SNP-Specific Associations in the Total Cohort

In the overall MASLD population, distinct single nucleotide polymorphisms (SNPs) demonstrated variable associations with clinical and biochemical traits relevant to liver disease progression (Table 3).

The PNPLA3 variant (analyzed under a dominant inheritance model) was significantly associated with elevated serum AST levels across the total cohort. Using partial Spearman correlations controlling for age and sex, also ALT levels resulted increased. This association was evident even among individuals with lower BMI, underscoring the variant’s robust effect on hepatocellular injury independent of adiposity.

The GCKR variant (recessive model) showed trends toward increased liver stiffness (LS; *p* = 0.071) and higher FIB-4 scores (*p* = 0.076), suggesting a possible role in promoting fibrosis, although these associations did not reach formal statistical significance.

Carriers of the MBOAT7 variant (recessive model) exhibited significantly higher CAP values, indicative of greater hepatic steatosis. Additionally, there was a trend toward elevated FIB-4 scores (*p* = 0.072), potentially linking this variant to both fat accumulation and fibrosis risk. This trend became significant (*p* = 0.035) after the analysis by partial Spearman correlations controlling for age and sex.

### 2.7. Cohort-Specific SNP Findings

#### 2.7.1. Cohort 1: Internal Medicine

In the internal medicine cohort, characterized by an older population with greater fibrosis burden, the impact of genetic variants was particularly pronounced (Table 3).

The PNPLA3 variant (dominant) was associated with higher AST and ALT levels, suggesting increased hepatocellular injury. A trend toward stronger associations with lower BMI was observed, reinforcing the notion that this variant exerts pathogenic effects beyond obesity alone.

The GCKR variant (recessive) was significantly associated with increased liver stiffness, pointing to a potential fibrogenic role in this metabolically vulnerable group.

The MBOAT7 variant (recessive) demonstrated a trend toward increased CAP values (*p* = 0.066), suggesting a contribution to hepatic fat accumulation in this cohort.

The TM6SF2 variant (dominant) showed trends toward elevated liver stiffness and higher AST levels, although these findings did not reach statistical significance. Nonetheless, they align with the variant’s known effects on hepatic lipid retention and liver injury.

#### 2.7.2. Cohort 2: Bariatric Surgery

In the bariatric surgery cohort, defined by younger age and more pronounced obesity and steatosis, distinct SNP associations emerged (Table 3).

The MBOAT7 variant (recessive) was significantly associated with increased AST and ALT levels, highlighting a potential role in hepatocellular injury within this population despite ongoing weight-loss interventions.

The TM6SF2 variant (dominant) was associated with reduced serum total cholesterol and triglyceride levels, consistent with its known impact on lipid metabolism and impaired hepatic lipid export. This effect may serve as a protective mechanism against systemic hyperlipidemia but at the cost of intrahepatic fat accumulation.

## 3. Discussion

In this cross-sectional study of 298 patients with MASLD, we investigated the interplay between genetic risk, clinical biomarkers, and non-invasive fibrosis metrics in two distinct cohorts: patients from a hepatology clinic and individuals undergoing bariatric surgery.

Consistent with previous literature, the polygenic risk score (PRS), incorporating variants in PNPLA3, TM6SF2, MBOAT7, and GCKR, demonstrated strong associations with hepatic injury markers (AST, ALT) and fibrosis risk (FIB-4). Importantly, patients in the highest PRS quartile or those exceeding the >0.532 threshold exhibited significantly higher AST and ALT levels and a trend toward increased fibrosis burden. These associations were particularly robust in the hepatology cohort, where genetic risk significantly correlated with both liver enzymes and fibrosis indices.

Notably, our data reinforce the predictive value of a PRS integrating *PNPLA3*, *TM6SF2*, *MBOAT7*, and *GCKR*, as previously proposed [12]. The association with FIB-4 even in a metabolic population underscores the PRS’s utility in early fibrosis detection.

SNP-level analysis provided further granularity. Variants in PNPLA3, GCKR, MBOAT7, and TM6SF2 were variably associated with liver injury and steatosis markers, with cohort-specific effects. Notably, PNPLA3 was associated with elevated transaminases even at lower BMI, consistent with its role as a key risk allele independent of adiposity. TM6SF2 was linked to lower lipid levels in the bariatric surgery cohort, supporting its known effects on hepatic lipid handling. MBOAT7 (rs641738), evaluated under a recessive model, was linked to higher CAP values and showed a trend toward greater fibrosis, suggesting a role in lipid remodeling and steatosis-driven fibrosis progression. In the hepatology cohort, GCKR (rs1260326) was associated with increased liver stiffness, possibly through altered glucose and lipid flux.

This study reveals distinct clinical and genetic profiles between MASLD patients from internal medicine and bariatric surgery settings. Despite lower BMI, patients in Cohort 1 exhibited significantly higher levels of liver enzymes and fibrosis markers (FIB-4, AST, ALT), indicating more advanced hepatic injury. These findings were accompanied by a higher polygenic risk score, suggesting a greater inherited predisposition to liver disease in this metabolically vulnerable group.

In contrast, Cohort 2, characterized by severe obesity, showed a higher tendency toward hepatic steatosis as measured by CAP, but lower levels of liver fibrosis and genetic risk. These differences highlight that severe obesity does not always correlate with more advanced liver damage and that genetic predisposition plays a pivotal role in disease severity, particularly in non-obese or less obese individuals. However, the mean LSM of 8.3 kPa suggests that we shouldn’t neglect this population for liver screening and, when appropriate, for hepatological evaluation.

These data suggest that in metabolically healthier, weight-loss-targeted populations, genetic risk may manifest predominantly through fat accumulation rather than fibrosis. Conversely, in hepatology patients, who may present at later disease stages, PRS more directly associates with fibrotic progression and liver injury.

Cohort-specific patterns underline how metabolic background modulates gene–phenotype interactions and underscore the importance of patient context in interpreting genetic effects. In internal medicine patients, transaminases tracked strongly with genetic risk, while in obese individuals, PRS was more closely aligned with steatosis (e.g., CAP) and dyslipidemia, potentially due to lower baseline fibrosis or shorter disease duration [17].

Single SNP analysis supports previous literature: *PNPLA3* as a major driver of liver injury, independently of obesity [18], and *TM6SF2* impacting lipid levels [19].

These findings support a model in which MASLD arises from the interaction of genetic and metabolic risk factors, with distinct manifestations in patients from different clinical settings. Incorporating PRS and SNP data into clinical evaluation could improve risk stratification and personalize surveillance and intervention strategies in MASLD.

## 4. Materials and Methods

### 4.1. Study Population

This study enrolled adult individuals diagnosed with metabolic dysfunction-associated steatotic liver disease (MASLD) at two tertiary care centers—the Hepatology Unit and the Bariatric Surgery Unit of the University Hospital “San Giovanni di Dio Ruggi d’Aragona”—over a two-year period. A total of 298 patients were included: 148 from the Hepatology Unit (Cohort 1) and 150 undergoing bariatric surgery (Cohort 2). All participants were of self-reported European ancestry (almost all from Southern Italy), minimizing population stratification effects. The study was conducted according to standard clinical practice protocols and was approved by the local Ethics Committee (CEI Campania Sud IRB n.8/2018). Informed consent was obtained from all participants prior to enrollment.

Inclusion criteria comprised: a diagnosis of MASLD in accordance with current clinical guidelines (hepatic steatosis on imaging or histology associated with at least one cardiometabolic risk factor, CMRF) [16]; age ≥ 18 years; overweight or obesity status; and provision of written informed consent.

Exclusion criteria included: (1) decompensated cirrhosis; (2) active malignancies, including hepatocellular carcinoma (HCC), or other serious comorbid conditions limiting life expectancy; (3) active or prior high-risk alcohol consumption (defined as >20 g/day for females and >30 g/day for males); and (4) active viral hepatitis or any liver disease of non-metabolic etiology.

**Figure 4:**
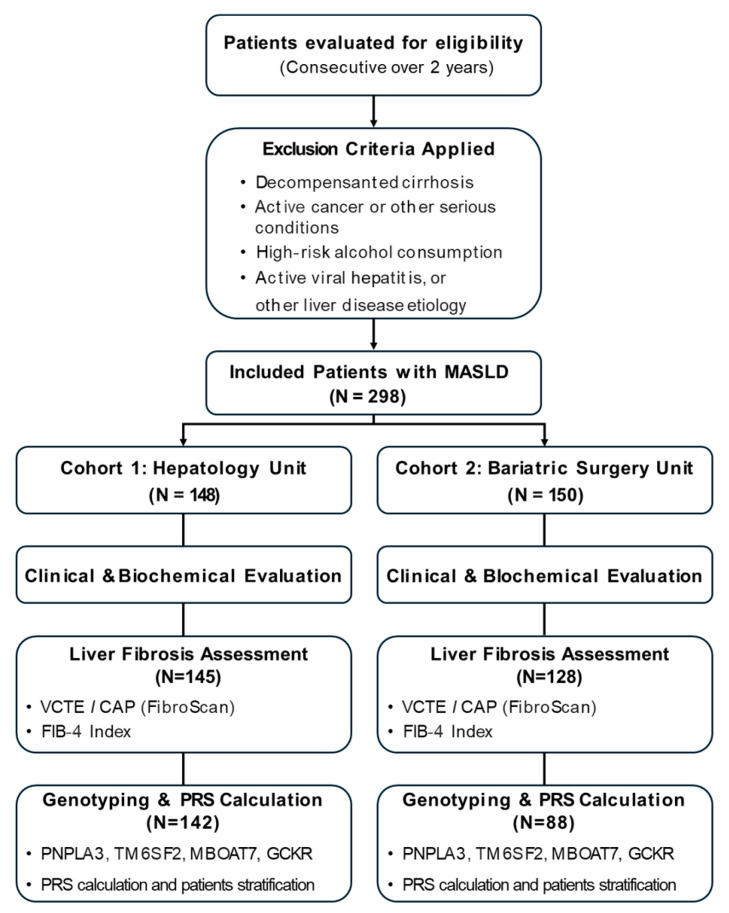
Study Design. Schematic representation of patient enrollment, cohort division (Hepatology vs. Bariatric), and assessment workflow including clinical evaluation, FibroScan^®^, genotyping, and PRS calculation.

A schematic study design is represented in Figure 4.

### 4.2. Clinical and Biochemical Assessment

All participants underwent a standardized clinical evaluation, including the collection of medical history, medication use, physical examination, and fasting blood sampling for biochemical analyses, routinely tested during a hospital visit. Qualified medical professionals collected blood samples for biochemical tests in the morning after overnight fasting into two polypropylene tubes. Two tubes (with a volume of 4 mL each) with a clotting activator were used to obtain blood serum, and another tube (with a volume of 4 mL) containing K_2_EDTA allowed the preparation of blood plasma and erythrocytes. 

### 4.3. Liver Fibrosis and Steatosis Assessment

Liver stiffness (LS) and steatosis were assessed by vibration-controlled transient elastography (VCTE) using the FibroScan^®^ Mini+ 430 device (Echosens, Paris, France), including measurements of controlled attenuation parameter (CAP). In addition, the Fibrosis-4 (FIB-4) index was calculated using the formula incorporating age, AST, ALT, and platelet count.

FIB-4 scores were stratified into three fibrosis risk categories:

- Low-risk: <1.3 (or <2.0 for individuals > 65 years);
- Intermediate risk: 1.3–2.67;
- High-risk: >2.67.

### 4.4. Genotyping and PRS

Genomic DNA was extracted from 200 µL of peripheral blood using the QIAamp DNA Mini Kit (Qiagen, Hilden, Germany) according to the manufacturer’s protocol. DNA quality and concentration were assessed using the Implen NP80 Touch spectrophotometer (Implen GmbH, Munich, Germany).

The *PNPLA3* rs738409, *TM6SF2* rs58542926, *MBOAT7* rs641738, and *GCKR* rs1260326 SNPs were selected as they are the most consistently replicated genetic variants influencing MASLD progression and have been included in clinically validated PRS models.

Genotyping of selected SNPs was performed via TaqMan^®^ 5′-nuclease allelic discrimination assays (ThermoFisher Scientific, Waltham, MA, USA). The following SNPs and inheritance models were used:

- PNPLA3 (rs738409): dominant model;
- TM6SF2 (rs58542926): dominant model;
- MBOAT7 (rs641738): recessive model;
- GCKR (rs1260326): recessive model.

A polygenic risk score (PRS) was computed as a weighted sum of risk alleles, reflecting each SNP’s reported effect size. PRS was stratified into quartiles, with a threshold of >0.532 defining the high-risk group.

### 4.5. Statistical Analysis

Continuous variables are expressed as mean ± standard deviation, and categorical variables as number (percentage). Group comparisons were assessed using the Kruskal–Wallis test for non-parametric data. Correlation analyses employed Spearman’s rho. Multivariate regression analyses were conducted to identify independent predictors of fibrosis. Statistical significance was defined as *p* < 0.05. Bonferroni correction was applied for multiple comparisons. For Table 1 (9 comparisons), α = 0.0056; for Table 2 (6 comparisons), α = 0.0083. The sample size required to detect a Spearman correlation of 0.300 with α = 0.05 and 80% power was ~140 participants per group, calculated using Fisher r-to-z transformation with variance adjustment [20]. We additionally conducted partial Spearman correlations controlling for age and sex to confirm observed associations. All analyses were performed using IBM SPSS Statistics for Macintosh, Version 26.0 (IBM Corp., Armonk, NY, USA).

## 5. Strengths and Limitations

This study benefits from a relatively large, well-characterized MASLD cohort with parallel data from both clinical hepatology and bariatric settings. Genotype data were analyzed under biologically informed models, and PRS construction accounted for weighted allele contributions. However, limitations include the cross-sectional design, limiting causal inference, and lack of histological data for liver fibrosis and steatosis confirmation. Additionally, unmeasured environmental and dietary factors may have influenced metabolic phenotypes. Further limitations include potential selection bias due to tertiary care-based recruitment, reducing generalizability to primary care settings. Confounding factors such as dietary patterns, physical activity, and concurrent medication use were not comprehensively adjusted.

## 6. Conclusions

Our findings confirm the clinical relevance of genetic variants and polygenic risk in shaping hepatic injury and steatosis profiles in MASLD. Incorporating PRSs into routine risk assessment may enhance early stratification and personalized management, particularly in heterogeneous populations such as those undergoing metabolic surgery versus those managed in hepatology clinics.

Future longitudinal studies are warranted to confirm these associations and evaluate the integration of genetic profiling into clinical management algorithms to enhance personalized care.

## Figures and Tables

**Table 1:** Characteristics of the Study Population.

Parameter	Total (N = 298)	Cohort 1 (n = 148)	Cohort 2 (n = 150)	*p*-Value
Age (years)	53.1 ± 13.1	58.3 ± 11.2	43.1 ± 10.4	2.7 × 10^−19^
Female	48.3%	41.9%	54.7%	0.027 #
BMI (kg/m^2^)	35.9 ± 6.4	33.1 ± 5.3	41.7 ± 4.6	3.9 × 10^−24^
AST (U/L)	34.3 ± 23.3	38.4 ± 26.6	25.8 ± 9.9	0.000013
ALT (U/L)	43.8 ± 33.4	48.3 ± 36.9	34.7± 22.1	0.005
FIB-4	1.75 ± 1.77	2.09 ± 1.98	1.02 ± 0.87	4.0 × 10^−5^
Liver Stiffness (kPa)	9.4 ± 10.2	10.3 ± 11.2	8.3 ± 7.9	NS
CAP (dB/m)	307.0 ± 42	302.0 ± 43.7	312.5 ± 39.4	NS
PRS	0.342 ± 0.23	0.368 ± 0.24	0.302 ± 0.21	0.047 #

Values are reported as mean ± standard deviation (SD) or percentage. Abbreviations: BMI (Body Mass Index), AST (Aspartate Aminotransferase), ALT (Alanine Aminotransferase), FIB-4 (Fibrosis-4 index), CAP (Controlled Attenuation Parameter), PRS (Polygenic Risk Score). # The unadjusted *p*-value was <0.05, but the association was not statistically significant after Bonferroni correction.

**Table 2:** Correlation matrix. Correlations between PRS, FIB-4, clinical parameters. Spearman’s ρ shown positive and significant correlations (*p* < 0.005).

			PRS	FIB-4	AST	ALT
**Spearman’s rho**	**PRS**	Correlation Coefficient	1.000	0.189 **	0.300 **	0.224 **
		Sig. (1-tailed)		0.004	0.000	0.001
		N	230	196	201	201
	**FIB-4**	Correlation Coefficient	0.189 **	1.000	0.552 **	0.174 **
		Sig. (1-tailed)	0.004		0.000	0.007
		N	196	203	203	203
	**AST**	Correlation Coefficient	0.300 **	0.552 **	1.000	0.822 **
		Sig. (1-tailed)	0.000	0.000		0.000
		N	201	203	208	208
	**ALT**	Correlation Coefficient	0.224 **	0.174 **	0.822 **	1.000
		Sig. (1-tailed)	0.001	0.007	0.000	
		N	201	203	208	208

Abbreviations: PRS (Polygenic Risk Score), FIB-4 (Fibrosis-4 index), AST (Aspartate Aminotransferase), ALT (Alanine Aminotransferase). Asterisks indicate significant correlations.

**Table 3:** SNP Associations with Liver Phenotypes (Spearman’s ρ, *p* < 0.05).

Variant	Associated Marker			Notes
	Total	Cohort 1	Cohort 2	
*PNPLA3*	AST ↑ BMI ↓	AST ↑ ALT ↑	-	Suggesting hepatic injury independently of adiposity
*GCKR*	LS and FIB-4 (*p* = 0.07)	LS ↑	-	Possible fibrosis impact through altered glucose and lipid flux
*MBOAT7*	CAP ↑	CAP (*p* = 0.066)	AST ↑ ALT ↑	Suggesting steatosis-driven injury
*TM6SF2*	-	-	TG ↓ Cholesterol ↓	Known cardioprotective/hepatotoxic profile

Abbreviations: *PNPLA3* (Patatin-like phospholipase domain-containing protein 3), *MBOAT7* (Membrane-bound O-acyltransferase domain-containing protein 7), *GCKR* (Glucokinase regulatory), *TM6SF2* (Transmembrane 6 superfamily 2), AST (Aspartate Aminotransferase), ALT (Alanine Aminotransferase), BMI (Body Mass Index), LS (Liver Stiffness), CAP (Controlled Attenuation Parameter), TG (Triglycerides).

## Data Availability

The data presented in this study are available on reasonable request.

## Abbreviations

The following abbreviations are used in this manuscript:

MASLD	Metabolic Dysfunction-Associated Steatotic Liver Disease
NAFLD	Non-Alcoholic Fatty Liver Disease (former term for MASLD)
PRS	Polygenic Risk Score
SNP	Single-Nucleotide Polymorphism
AST	Aspartate Aminotransferase
ALT	Alanine Aminotransferase
FIB-4	Fibrosis-4 Index
LS	Liver Stiffness
CAP	Controlled Attenuation Parameter
BMI	Body Mass Index
TG	Triglycerides
HCC	Hepatocellular Carcinoma
VCTE	Vibration-Controlled Transient Elastography
LSM	Liver Stiffness Measurement
CMRF	Cardiometabolic Risk Factor
SPSS	Statistical Package for the Social Sciences
PNPLA3	Patatin-like phospholipase domain-containing protein 3
MBOAT7	Membrane-bound O-acyltransferase domain-containing protein 7
GCKR	Glucokinase regulatory
TM6SF2	Transmembrane 6 superfamily 2
